# Spontaneous Intracranial Hypotension Complicated by Bilateral Cerebral Venous Thrombosis in an Older Adult: A Case Report

**DOI:** 10.7759/cureus.90590

**Published:** 2025-08-20

**Authors:** Md Zubiar Haque, Ganesh Arunachalam, Robert Ghosh

**Affiliations:** 1 Geriatrics, The Princess Alexandra Hospital NHS Trust, Harlow, GBR; 2 Internal Medicine, The Princess Alexandra Hospital NHS Trust, Harlow, GBR

**Keywords:** anticoagulation, cerebral venous sinus thrombosis (cvst), headache, orthostatic headaches, spontaneous intracranial hypotension (sih), subdural hygroma

## Abstract

Spontaneous intracranial hypotension (SIH) is a rare cause of headaches, sometimes presenting atypically in older adults. We report the case of a woman in her early 70s with chronic frontal headaches, gait imbalance, and transient hand weakness. Initial computed tomography (CT) of the head suggested sinusitis, but venous imaging revealed cerebral venous sinus thrombosis (CVST), prompting anticoagulation with rivaroxaban. Readmission for facial droop showed bilateral subdural hygromas. Multidisciplinary assessment identified SIH as the likely primary condition, with CVST as a complication. Anticoagulation was transitioned to warfarin. The patient recovered fully with resolved CVST on imaging. This case highlights the importance of early multidisciplinary collaboration and clinical vigilance in establishing a timely diagnosis and achieving a favourable outcome in suspected SIH.

## Introduction

Spontaneous intracranial hypotension (SIH) is an uncommon but increasingly recognised condition resulting from reduced cerebrospinal fluid (CSF) volume and pressure, most frequently due to a spontaneous spinal CSF leak [1]. The hallmark symptom is orthostatic headache, worsening when upright and improving when recumbent. Presentations can vary significantly, particularly in older adults, as seen in our patient [2]. Patients may also experience neck stiffness, nausea, cognitive disturbances, visual changes, or tinnitus, all of which contribute to diagnostic uncertainty and often mimic more common neurological conditions [3].

Magnetic resonance imaging (MRI) uses strong magnetic fields and radiofrequency waves to align and disturb the hydrogen nuclei of the water molecules of the body, then detects the emitted signal as they relax to create detailed images of tissues. Brain MRI is essential in evaluating suspected SIH. Typical findings include pachymeningeal enhancement (diffuse thickening and enhancement of the dura on MRI), brain sagging, and subdural collections, with a sensitivity of approximately 83% for detecting at least one imaging sign of SIH [4]. The Bern score provides a structured framework for radiological diagnosis; however, SIH should not be excluded based on the absence of typical features like orthostatic headache or abnormal imaging [5].

Complications of SIH, while rare, can be significant. These include cranial nerve palsies, subdural hygromas or haematomas, and in some cases, cerebral venous sinus thrombosis (CVST), which may result from venous stasis due to intracranial hypovolemia [6]. Recognising such complications early is crucial, as they may dominate the clinical picture and obscure the underlying diagnosis.

This report describes a rare presentation of SIH in an elderly patient with bilateral CVST and subdural hygromas. It highlights the diagnostic challenges in atypical cases and reinforces the importance of early imaging, multidisciplinary collaboration, and consideration of SIH in the differential diagnosis of unexplained chronic headaches with vascular complications.

This case report's abstract has been accepted for a virtual poster presentation at the International Conference on Neurology and Brain Disorders (INBC 2025), scheduled to take place from 20 to 22 October 2025.

## Case presentation

A woman in her early 70s presented with chronic, intermittent frontal headaches that had persisted for over six months. The headaches were non-radiating, without clear aggravating or relieving factors, and were initially responsive to over-the-counter analgesics, including paracetamol and ibuprofen. She also reported feeling generally unwell, with occasional gait imbalance and intermittent hand weakness. There was no history of trauma, nausea, loss of consciousness, seizures, or sensory disturbances. Her past medical history included hypertension, osteoarthritis, and gastritis. She was self-caring and independent in activities of daily life.

**Figure 1:**
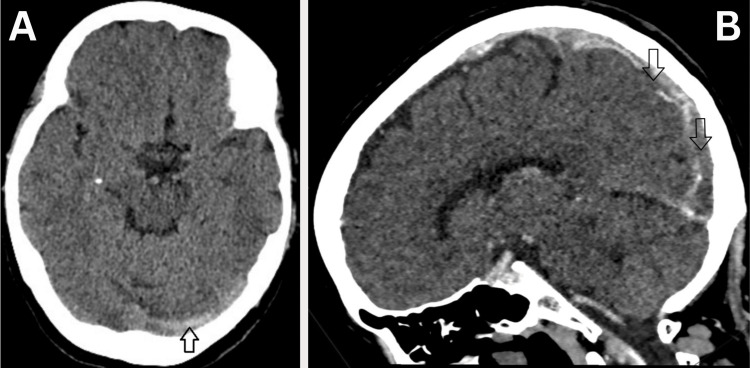
(A) Axial non-contrast CT head demonstrating hyperdensity within the superior sagittal sinus and cortical veins (arrow), consistent with acute cerebral venous sinus thrombosis (CVST). (B) Sagittal CT venogram showing filling defects in the superior sagittal sinus (arrows), consistent with CVST.

Initially, her symptoms were attributed to recurrent sinusitis, and she received multiple courses of antibiotics in the community. However, the persistence of symptoms led her general practitioner to request a non-contrast CT scan of the brain, which was reviewed in hospital, and revealed hyperdensity in the superior sagittal sinus and cortical veins, findings suggestive of acute CVST (Figure 1A). On admission, she was asymptomatic. Her vital signs, including blood pressure and temperature, were within normal limits. Physical examination revealed no focal neurological deficit. Subsequent CT venography confirmed the presence of bilateral CVST (Figure 1B). In the absence of relevant symptoms, she was discharged on a direct oral anticoagulant, rivaroxaban.

**Figure 2:**
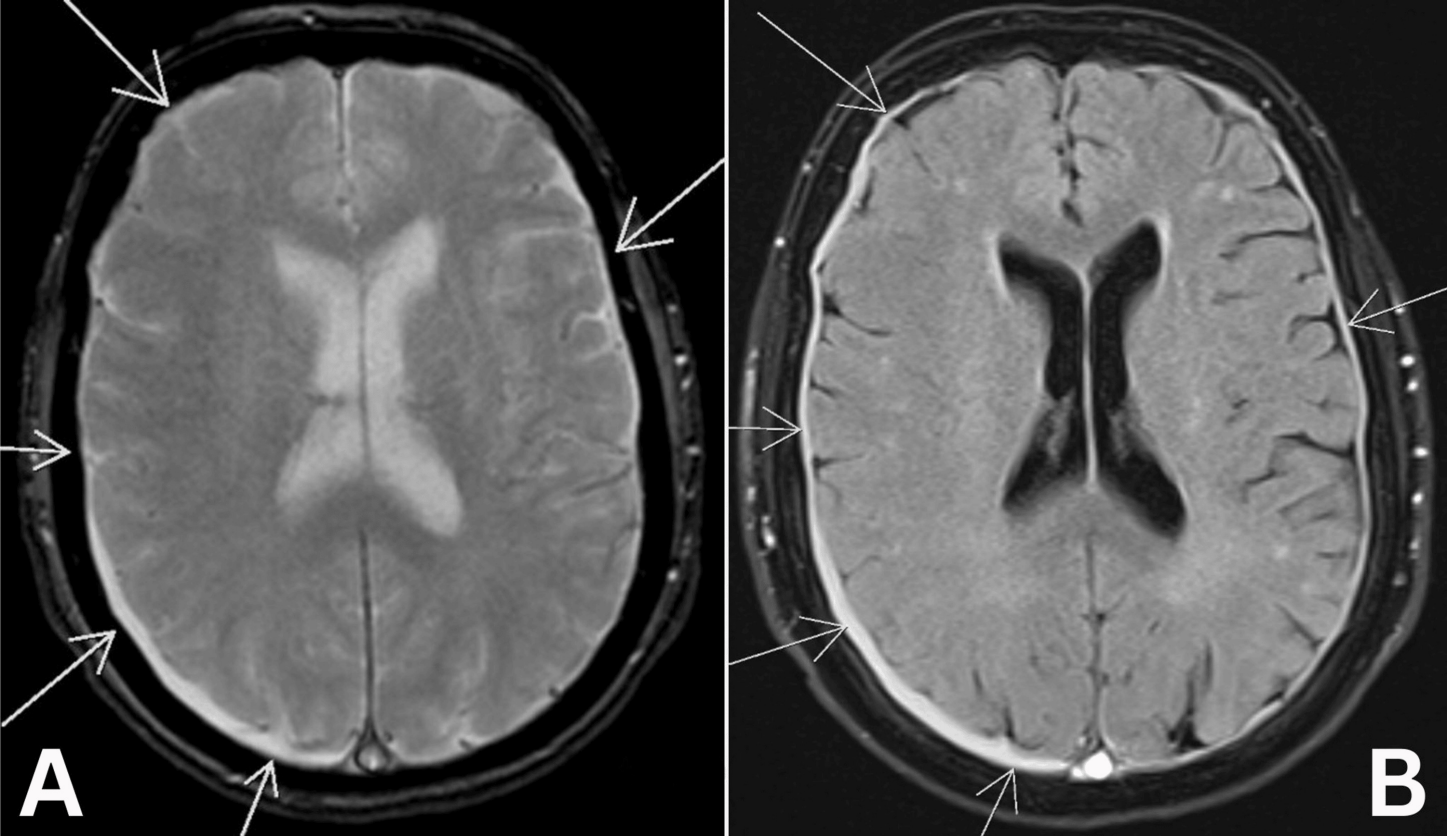
Axial MRI head images demonstrating bilateral subdural hygromas. (A) T2*-weighted gradient echo sequence showing bilateral subdural collections (arrows), more prominent on the right. (B) Fluid-attenuated inversion recovery (FLAIR) sequence showing the same subdural collections (arrows).

The patient was readmitted after eight days with a transient episode of right-sided facial droop, which resolved spontaneously prior to hospital attendance. Physical examination, including a detailed neurological assessment, was normal. MRI of the brain demonstrated extensive CVST and bilateral shallow subdural collections, more prominent on the right, consistent with subdural hygromas (Figure 2). Gradient echo sequences ruled out acute intracranial haemorrhage (Figure 2A). Further diagnostic workup for the facial droop, including carotid Doppler ultrasound and electroencephalography (EEG), was unremarkable, effectively excluding transient ischaemic attack (TIA) and epileptic disorder. The episode was attributed to venous ischaemia related to her CVST.

A multidisciplinary team consisting of general medicine, radiology, haematology, and neurology specialists reviewed her case and concluded that the subdural collections represented hygromas rather than haemorrhages, making it safe to continue anticoagulation. Given the imaging findings and the absence of other risk factors, SIH was considered the likely underlying aetiology for her clinical presentation. Following haematology review, a thrombophillia screen was performed and was negative. Also, her anticoagulation was transitioned from a direct oral anticoagulant (DOAC) to warfarin.

**Figure 3:**
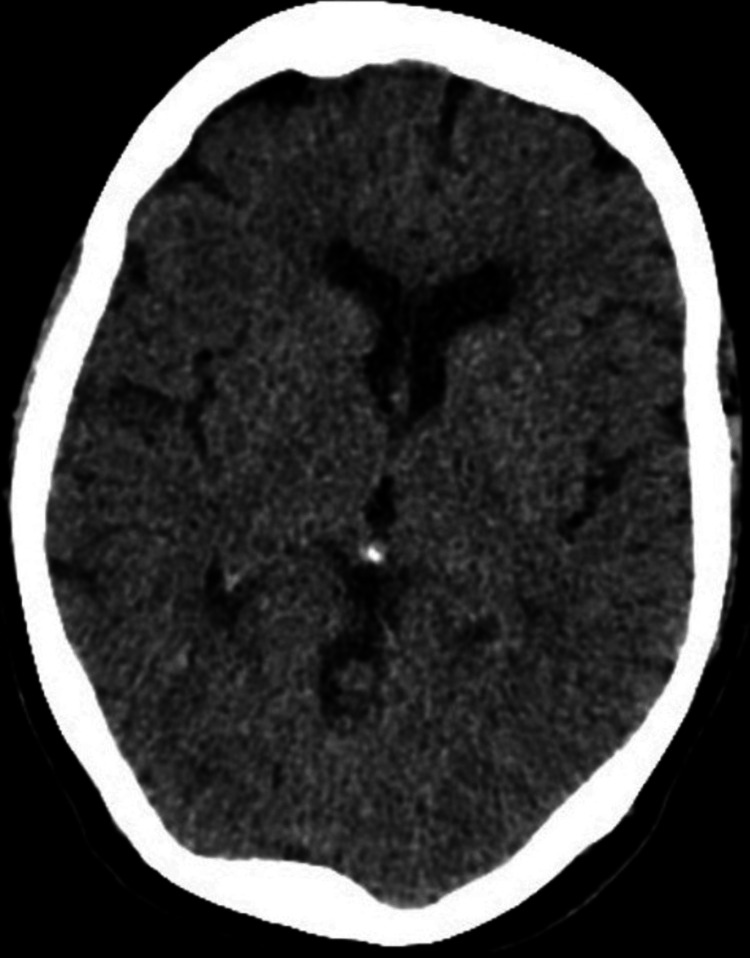
Axial non-contrast CT head showing complete resolution of cerebral venous sinus thrombosis (CVST).

At follow-up, she remained asymptomatic and demonstrated no further neurological symptoms, including headaches, visual disturbances, or speech problems. Repeat CT imaging confirmed near-complete resolution of the venous thrombosis (Figure 3), and she continues with anticoagulation under outpatient supervision.

## Discussion

SIH is an uncommon clinical entity that results from a spontaneous CSF leak, typically at the spinal level, leading to decreased intracranial CSF volume and pressure. Although the incidence is estimated at five per 100,000 per year, it is widely believed to be underdiagnosed due to its heterogeneous and sometimes subtle clinical manifestations [5].

The hallmark symptom of SIH is orthostatic headache, characterised by worsening pain upon standing and relief with recumbency [2]. However, not all patients present with this classic feature. Additional symptoms can include neck stiffness, nausea, visual or auditory disturbances, and cognitive changes, which can mimic other conditions such as migraine, sinusitis, or TIA [3]. This variability often leads to misdiagnosis or delayed identification of the underlying pathology.

In the current patient, the absence of orthostatic headache and the presence of neurological signs, including transient facial droop and hand weakness, contributed to diagnostic complexity. Eventual CT venography revealed bilateral CVST, a rare but increasingly recognised complication of SIH [6]. The exact mechanism linking SIH to CVST is not fully established, but it is hypothesised that low CSF pressure results in compensatory venous engorgement and stasis, predisposing patients to thrombosis formation [7]. Although spinal imaging or lumbar puncture was not performed, the constellation of findings strongly supported a diagnosis of SIH in this patient. 

Neuroimaging is crucial for diagnosing SIH and differentiating it from other intracranial pathologies. In the present patient, MRI findings of bilateral subdural hygromas, likely a direct consequence of CSF loss and absence of haemorrhage on gradient echo sequences, supported a diagnosis of SIH, despite incomplete fulfilment of the Bern score [4,8]. The Bern score is a validated radiological tool for diagnosing SIH based on characteristic MRI findings, though atypical cases may fall outside its criteria. 

Management of SIH typically includes conservative measures such as bed rest, hydration, caffeine, and, when needed, an epidural blood patch [9]. In patients with SIH complicated by CVST, anticoagulation remains essential. In our patient, advanced imaging confirmed the absence of haemorrhage, allowing safe continuation of anticoagulation. The patient responded well to therapy and remained stable on follow-up.

This unusual presentation in this patient highlights the diagnostic challenge SIH poses when classic features are absent. It underscores the importance of a high index of suspicion and the role of multidisciplinary collaboration. Clinicians should consider SIH in patients with unexplained chronic headaches, particularly when neuroimaging reveals subdural collections or CVST without traditional risk factors. Furthermore, awareness of complications such as CVST is essential, as these may dominate the clinical picture and lead to delayed diagnosis of the underlying cause. CVST-associated mortality rate ranges from approximately 4% to 13%, with many cases resulting in incomplete recovery [10]. Therefore, it remains essential that patients with CVST and SIH undergo follow-up, combining clinical and neuroradiological assessment.

## Conclusions

This case highlights the importance of considering SIH in older adults presenting with chronic or atypical headaches, particularly when imaging reveals bilateral subdural collections or cerebral venous sinus thrombosis. SIH may present without classic orthostatic features, and its complications, such as CVST, can dominate the clinical picture. Advanced imaging is essential for differentiating subdural hygromas from haemorrhage, allowing for safe management. Early multidisciplinary involvement supports accurate diagnosis and management. Clinicians should maintain a high index of suspicion and work to exclude secondary causes of CVST.
